# Multi-Parametric MRI at 14T for Muscular Dystrophy Mice Treated with AAV Vector-Mediated Gene Therapy

**DOI:** 10.1371/journal.pone.0124914

**Published:** 2015-04-09

**Authors:** Joshua Park, Jacqueline Wicki, Sue E. Knoblaugh, Jeffrey S. Chamberlain, Donghoon Lee

**Affiliations:** 1 Department of Radiology, University of Washington, Seattle, Washington, United States of America; 2 Department of Neurology, University of Washington, Seattle, Washington, United States of America; 3 Comparative Medicine Shared Resources, Fred Hutchinson Cancer Research Center, Seattle, Washington, United States of America; 4 Department of Biochemistry, University of Washington, Seattle, Washington, United States of America; 5 Department of Medicine, University of Washington, Seattle, Washington, United States of America; Earl and Christy Powell University, UNITED STATES

## Abstract

The objective of this study was to investigate the efficacy of using quantitative magnetic resonance imaging (MRI) as a non-invasive tool for the monitoring of gene therapy for muscular dystrophy. The clinical investigations for this family of diseases often involve surgical biopsy which limits the amount of information that can be obtained due to the invasive nature of the procedure. Thus, other non-invasive tools may provide more opportunities for disease assessment and treatment responses. In order to explore this, dystrophic *mdx^4cv^* mice were systemically treated with a recombinant adeno-associated viral (AAV) vector containing a codon-optimized micro-dystrophin gene. Multi-parametric MRI of T2, magnetization transfer, and diffusion effects alongside 3-D volume measurements were then utilized to monitor disease/treatment progression. Mice were imaged at 10 weeks of age for pre-treatment, then again post-treatment at 8, 16, and 24 week time points. The efficacy of treatment was assessed by physiological assays for improvements in function and quantification of expression. Tissues from the hindlimbs were collected for histological analysis after the final time point for comparison with MRI results. We found that introduction of the micro-dystrophin gene restored some aspects of normal muscle histology and pathology such as decreased necrosis and resistance to contraction-induced injury. T2 relaxation values showed percentage decreases across all muscle types measured (tibialis anterior, gastrocnemius, and soleus) when treated groups were compared to untreated groups. Additionally, the differences between groups were statistically significant for the tibialis anterior as well. The diffusion measurements showed a wider range of percentage changes and less statistical significance while the magnetization transfer effect measurements showed minimal change. MR images displayed hyper-intense regions of muscle that correlated with muscle pathology in histological sections. T2 relaxation, alongside diffusion and magnetization transfer effects provides useful data towards the goal of non-invasively monitoring the treatment of muscular dystrophy.

## Introduction

Muscular dystrophy is a group of inherited diseases which are characterized by progressive muscle weakness that over time leads to muscle damage and wasting [1]. Duchenne muscular dystrophy (DMD) is among the most commonly occurring forms of muscular dystrophy affecting approximately 1 in every 3,600 male infants. The disease is recessively X-linked, meaning while females may carry the genes responsible for the mutation, only males will express the phenotype, thus leading to the onset of DMD [2]. The disease is caused by a mutation in the dystrophin gene leading to abnormal or absent dystrophin protein which is responsible for linking actin filaments in muscle fiber to support proteins within the plasma membrane [3, 4]. Without dystrophin, muscle fibers slowly experience damage that eventually leads to necrosis and replacement of muscle fibers by fatty and connective tissue [5]. This progressive muscle deterioration ultimately culminates in the failure of the heart and/or the muscles responsible for respiration, leading to death. There is no cure and the average life expectancy of an afflicted individual is 25 years [6].

Gene therapy for muscular dystrophy is a promising treatment strategy because it has the potential to restore dystrophin expression in dystrophic muscle, and thereby improve muscle function and stabilize disease progression in all DMD patients regardless of the nature of their genetic mutations. Recombinant adeno-associated viral (rAAV) vectors have become the vector of choice because they are not known to cause any human disease and are efficient in transducing skeletal and cardiac muscles. However, the small packaging capacity of rAAV vectors prevents the insertion of a large dystrophin transgene. To overcome this issue, nonessential portions of dystrophin, some originally identified from studies of mild Becker muscular dystrophy (BMB) patients, were removed to develop functional micro-dystrophins (μDys). A number of these micro-dystrophin proteins have been shown to restore the dystrophin-associated protein complex, stabilize muscle degeneration, and improve muscle function following AAV-mediated gene delivery in animal models of DMD including *mdx* mice and dystrophin/utrophin double knockout mice at different stages of disease progression [7–17]. Pacak *et al*. demonstrated favorable responses including T2 MRI against AAV-mediated gene therapy for a mouse model of limb girdle muscular dystrophy [18]. Furthermore, muscle-specific promoters have been developed to restrict expression to target muscle cells and potentially reduce the possibility of an undesirable immune response [19]. However, regardless of the delivery methods and levels of transduction, micro-dystrophin proteins do not fully protect dystrophic muscle from contraction-induced injury [12, 14–16].

The animal model most commonly used to investigate therapeutic approaches for Duchenne muscular dystrophy is the *mdx* mouse. The *mdx* mouse undergoes a critical period between weeks 4 and 5 during which there is a peak in muscle degeneration and regeneration. This phase is followed by a gradual decrease in necrosis until a low level is reached in adult mice at around 3–4 months of age. Although the *mdx* phenotype is much less severe than that seen in older DMD patients, certain aspects of DMD (elevated CK, centrally nucleated muscle fibers, variations in cell size, susceptibility to contraction-induced injury) are more closely reproduced in muscle tissues of adult *mdx* mice than younger *mdx* mice. Other aspects of DMD pathology are not well modeled by the *mdx* mouse (fat infiltration and fibrosis). Endpoints for evaluation of treatment traditionally include histological analyses of muscle sections to quantify the area of damage, fiber size and/or fibrosis, blood parameters (creatine kinase, CK), and muscle force/function.

Magnetic Resonance (MR) has emerged as an alternative tool for gathering valuable information pertaining to tissue characteristics. Historically, such information was acquired via surgical biopsy—however, the invasive nature of the procedure and the limited sampling regions greatly restricted the amount of information attained surrounding the dysfunction [20–26]. However, recently developed multimodal MR approaches have extended evidence that MR can significantly facilitate noninvasive diagnosis and monitoring of muscle dysfunction [26]. Magnetic resonance imaging (MRI) can be used to detect pathological changes in skeletal muscle at both the cellular and tissue level [27, 28]. It has been demonstrated that differences and abnormalities in muscle integrity could be detected in cardiac muscle of *mdx* mice as early as one month of age [29]. Multi-parametric MRI of T1, T2, magnetization transfer (MT), and diffusion effects have been shown to be feasible identifiers of tissue and cellular necrosis along with regeneration in skeletal muscles [26]. Similar studies suggest quantitative muscle MR is an important longitudinal outcome measure for the assessment of muscle pathology and monitoring of therapeutic efficacy [30]. Additionally, molecular alterations detected by MR can be compared to distinct histologic changes in dystrophic tissues [31–33].

In this study, we conducted longitudinal magnetic resonance imaging at 14 Tesla (T) utilizing the T2 relaxation, diffusion, and MT modalities in conjunction with high resolution 3-D (dimensional) imaging and histological analysis to track and evaluate the efficiency and sensitivity of MR as a non-invasive tool for the monitoring of disease progression and treatment responses for a mouse model (*mdx* ^*4cv*^) of muscular dystrophy. The efficacy of treatment was evaluated at the endpoint of the experiment using standard methods. The approaches included histological analysis of myofibers and immunofluorescence (IF) to confirm the extent of micro-dystrophin expression, alongside measurements of specific force and resistance to contraction-induced injury to evaluate function.

## Materials and Methods

### Mice

There were 7 B6Ros.Cg-*Dmd* ^*mdx-4Cv*^/J (abbreviated *mdx* ^*4cv*^ or *mdx*) mice [34], along with 3 age matched normal C57BL/6J mice, that were 10 weeks of age when used for this study. All animals were housed and treated in strict accordance with the National Institutes of Health (NIH) Guide for the Care and Use of Experimental Animals and approvals from the Institutional Animal Care and Use Committee (IACUC, protocol number: 4210–01 and 3333–01) of the University of Washington. The mice were housed in SPF (specific pathogen free) facilities running 12:12 light/dark cycles at ambient temperatures of 22–23°C. The mice had access to food and water *ad libitum*. These conditions were maintained throughout the duration of the study. Mice were imaged under isoflurane anesthesia and limited in prolonged imaging sessions to minimize animal suffering. For the histological and functional measures, mice were anesthetized intraperitoneally with Avertin (0.5 mg/g body weight) with supplemental doses as required to maintain necessary anesthesia, then euthanized by cervical while still under anesthesia. All mice were euthanized following the conclusion of the study.

### Generation of Constructs, Vector Production and Delivery

A codon-optimized version of micro-dystrophin (ΔR4-23/ΔCT) cDNA [35] was designed and synthesized by GenScript (Piscataway, NJ). The optimized cDNA sequence was cloned into an AAV expression plasmid containing serotype 2 inverted terminal repeats, a muscle-specific promoter (CK8) based on enhancer/promoter regions of the murine muscle creatine kinase gene, and a highly efficient synthetic poly(A) signal as previously described [15]. The resulting construct, CK8.H3μDys, was sequenced and then co-transfected with the pDGM6 packaging plasmid into HEK293 cells to generate a recombinant AAV6 vector that was harvested and purified on a heparin column [14]. Southern analysis was utilized to determine the number of genome-containing particles in the vector preparation [12]. Four of the 7 *mdx* mice were systematically treated with 10^13^ vector genomes of rAAV6.CK8.H3μDys vector by retro-orbital (RO) injection at 3 months of age while the remaining 3 acted as untreated controls.

### MRI

Multi-parametric ^1^H MRI was carried out for *mdx* ^*4cv*^ mice on a Bruker 14T Avance MR spectrometer (Bruker Corp., Billerica, MA). As summarized in Table 1, the high resolution MRI protocol includes scout imaging (gradient echo; TR (repetition time)/TE (echo time) = 30/1.3 ms), planning for image planes (multi-slice RARE (rapid acquisition with refocused echoes): TR/TE = 668/4.5 ms), high resolution 2 dimensional imaging with 55 thin slices (200 micron thick) (multi-slice RARE: TE/TE = 4000/6 ms) for muscle volume evaluation, multi-slice multi-echo imaging (TR/TE = 4000/ 6 ~ 100 ms, 16 echoes with 6.3 ms spacing) for transverse relaxation time T_2_ measurements, magnetization transfer imaging (gradient echo; TR/TE = 939/5 ms, flip angle = 30°), diffusion imaging with three b values of ~25, 586 and 1111 s/mm^2^ sequence (TR/TE = 3751/27.5 ms). Table 1 also describes a goal of each MR method relating with muscular dystrophy.

**Table 1:** Mouse MR Protocols.

Method	Sequence type	TR/TE (ms)	Acq. Time	Purpose
Scout imaging	GRE	30/1.3	~ 5 sec	3 orthogonal images
Image planning	GRE	668/4.5	~ 1 min	Slice position and orientation selected
T2	MSME	4000/6;16 echoes	9 min	T2 maps to visualize T2 changes between normal and affected regions
Diffusion	SE	3751/27.5, b = 25, 586, 1111 s/mm^2^	25 min	ADC maps to visualize changes with edema and myocyte damage [49, 50]
MT	GRE	939/5 ms, θ = 30° for with and without MT effect	~ 5 min	MT Ratio maps to emphasize macromolecular changes [51–54]; to visualize fibrosis [55, 56]
3D imaging	GRE	100/3, θ = 10°	~ 5 min	Visualize and measure muscle volumes

TR: recycle delay, TE: echo time, GRE: gradient echo; SE: spin echo; MSME: multi-slice multi-echo; θ: Flip Angle

The quantitative T2 measurements utilized spin echo sequences to generate T2 maps—T2 maps were generated using a multi-slice multi-echo sequence (TR/TE = 4 s/ 6 ~ 100 ms, 16 echoes) with fat signal suppressed (gaussian pulse, pulse length = 1.3 ms, bandwidth = 2100.5 Hz) at 14T. We utilized: $SI={Ae}^{-\frac{TE}{T2}}$ to fit the T2 values in order to generate maps, where *SI* is the signal intensity and A is the amplitude. T2 weighted images were used to not only visually inspect the muscles for apparent signs of necrosis and damage, but also to qualitatively measure comparable regions of interest to detect changes between muscles and time points. These images were used to longitudinally compare both the treated/untreated *mdx* ^*4cv*^ mice and the control mice.

*In vivo* magnetic resonance imaging can be sensitized to local characteristics of water diffusion by utilizing the Brownian characteristic of water molecules [36–38]. Apparent Diffusion Coefficient (ADC) maps were generated by carrying out diffusion weighted imaging utilizing the three b values of ~25, 586 and 1111 s/mm^2^. The gradients incorporated a spin echo pulse sequence (TR/TE = 3751/27.5 msec). The Stejskal-Tanner spin echo pulse sequence was used with different pulses of 7 ms (pulse duration) with 14 ms (pulse interval) between the gradient pulses. These diffusion gradients were oriented along the direction of the slice section gradient. The function: *b* = *s*_0_ *e* ^−b*ADC^, where *s*_0_ is the intensity with no diffusion gradient (b = 0), was used likewise to the T2 in order to generate maps containing ADC values.

Magnetization Transfer (MT) suppression ratios, or MT ratios (MTRs), were measured using the following ratio: (*SI*_*o*_ – *SI*_*s*_)/*SI*_*o*_, where *SI*_*o*_ represents the tissue signal intensity with no saturation pulse applied while *SI*_*s*_ includes the saturation pulse. We utilized a gradient echo sequence (TR/TE = 939/5 ms, flip angle = 30°) with an off-resonance frequency of 5000 Hz and a saturation pulse of block pulse shape, 50 msec width, and 10 μT amplitude.

All *mdx* ^*4cv*^ were imaged at 10–11 weeks of age for the initial pre-treatment (Pre-Rx) time point and then subsequently imaged at 8, 16, and 24-week time points post gene therapy treatment (Post-Rx) to monitor disease/treatment progression. The 3 normal control C57Bl/6J mice were imaged at 10–11 weeks of age as well and then re-measured during the 24-week time point. Mice were induced using 5% isoflurane in oxygen and then maintained at a lower rate of 1.5% isoflurane in oxygen throughout the imaging process. During this time, the mice were monitored to not only preserve mouse condition, but also minimize motion effects in the imaging process via image gating. This was accomplished by using the MR-compatible small animal monitoring and gating system (SA Instruments Inc., NY, USA). The system monitors and accounts for the movement that is associated with animal respiration thus allowing us to remove motion artifacts from the MR images by triggering during the MR acquisition.

### Image Analysis

MRI images were analyzed using ImageJ software (http://rsbweb.nih.gov/ij), developed by the National Institutes of Health, to measure mean values of particular muscles including tibialis anterior (TA), gastrocnemius (GA), and soleus (SOL). Regions of interest (ROIs) were selected to be consistent in size and location across 5 consecutive slices, avoiding hyper-intensive regions (representing necrotic areas or regions of inflammation/edema), on each leg. ROIs were circular and spanned an area of 80 pixels or 8 mm^2^. Total muscle volumes, as well as hyper-intensive region volumes, were measured using Amira (Visualization Sciences Group, Burlington, MA), a 3-D software platform. 3-D reconstructions of both muscle and hyper-intensive necrotic regions were also completed using Amira.

### Statistical Analysis

PRISM version 6 software (GraphPad Software, USA) and the two-way analysis of variance (ANOVA) were used for statistical analyses for the following time points: (Pre-Rx, 8 week (wk) Post-Rx, 16 wk Post-Rx, and 24 wk Post-Rx). For the ANOVA, we used the Tukey’s multiple comparisons test for the data with the significance of 0.05 and confidence interval of 95% to generate P values.

### Measurement of Serum Creatine Kinase Levels

Blood samples were collected via retro-orbital bleeds at the experimental endpoint under anesthesia. Whole blood was allowed to clot for a minimum of 30 minutes in BD Microtainer Tubes (BD Diagnostics, Franklin Lakes, NJ) and was then centrifuged for 90 seconds at 12,000 g. Measurement of the serum creatine kinase activity was performed by Phoenix Central Laboratories (Mukilteo, WA).

### Muscle Physiology

Following completion of the MR study, contractile properties were measured *in vitro* for force generation and protection from contraction-induced injury using methods previously described [39]. The mice were anesthetized with an intraperitoneal injection of 2,2,2-tribromoethanol (Sigma Aldrich, St. Louis, MO), with supplemental injections as required to prevent response to tactile stimuli. Extensor digitorum longus (EDL) muscles were isolated and proximal and distal tendons were tied firmly with 6–0 silk sutures. One end of the tendon was tied to a force transducer and the other tendon to the lever arm of a servomotor (Aurora Scientific, Aurora, ON, Canada). Muscles were stimulated directly with a pulse duration of 2 ms. With the muscle length set at optimum length (*L*_o_) the maximum isometric tetanic force (*P*_o_) was determined. The susceptibility of EDL muscles to contraction-induced injury was assessed by seven lengthening contractions. The muscles were set at *L*_o_, activated maximally, and then stretched through a strain of 30%, relaxed for a 30 s recovery period and then exposed to multiple stretches of 30%. Following contractile evaluation, muscles were weighed to calculate specific force. The total fiber cross-sectional area (CSA) was calculated based on the measurements of optimal muscle length (mm), muscle mass (mg), a muscle density of 1.06 g/cm^2^ and an *L*_f_/*L*_o_ ratio of 0.44 for the EDL muscles [39]. The specific force *sP*_o_ (kN/m^2^) was determined by dividing *P*_o_ (kN) by the CSA (m^2^). The force deficit produced by the lengthening contraction protocol (LCP) was assessed by expressing the maximum isometric force *P*_o_ (mN) measured after the lengthening contraction protocol as a percentage of the *P*_o_ (mN) before injury. After being subjected to functional testing, the mice were euthanized and individual tissues from the right hindlimb were rapidly excised and processed for IHC and histological analysis. The left hindlimbs were amputated and fixed in 10% neutral buffered formalin for subsequent paraffin section preparation.

### Histology

**Fig 1:**
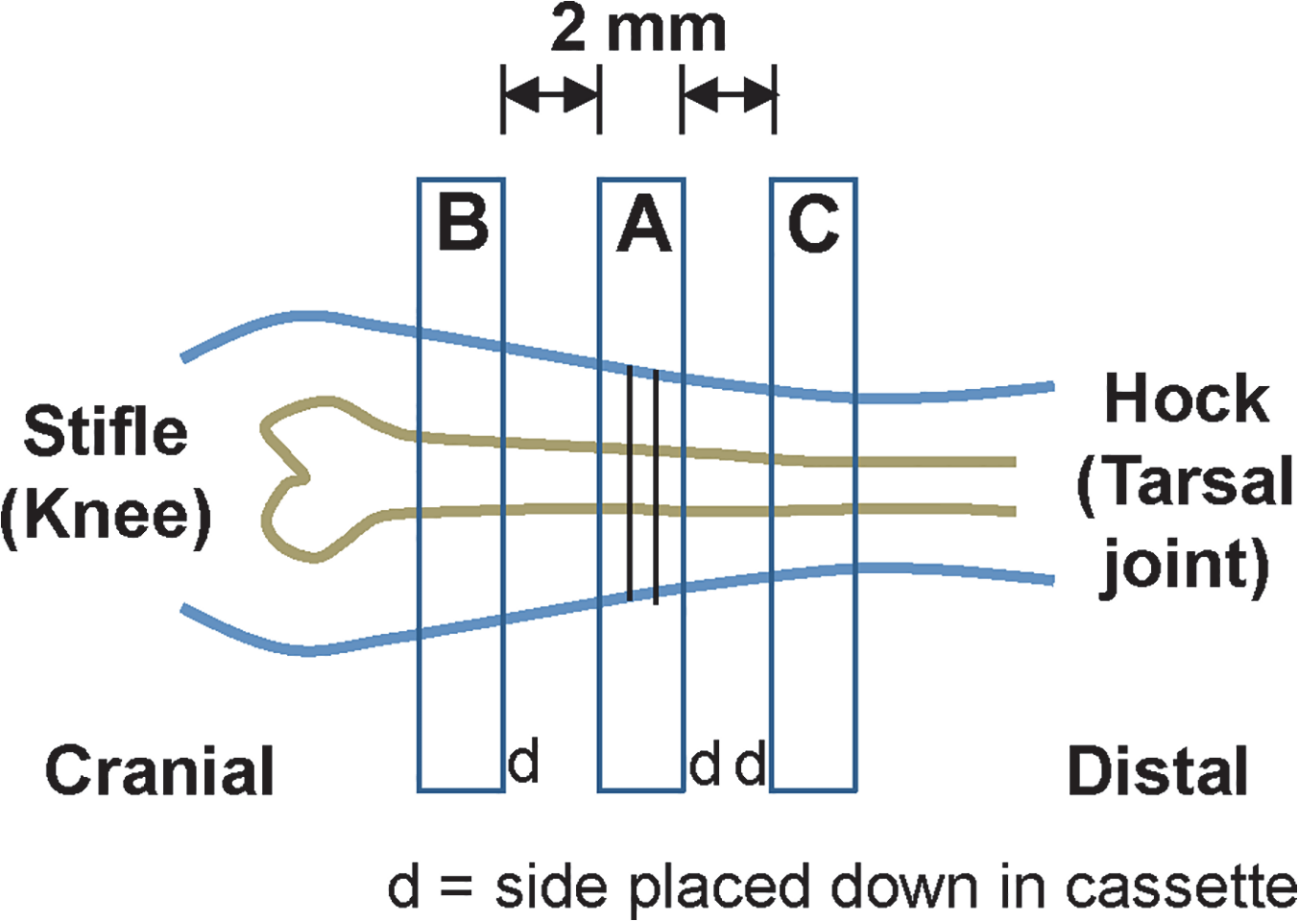
Sectioning scheme for histology results.

In order to compare MRI results with histopathology, the left hindlimbs were collected and fixed in 10% neutral buffered formalin for 5–7 days. The samples were decalcified with Rapid-Cal Immuno, from BBC biochemical (http://www.bbcus.com, Seattle, WA). Areas of interest were based on MRI results and were measured in relation to the patella. Tissue marking ink was used to create a 2–3 mm wide external mark circumscribing the tibia, fibula, and associated muscles. Hindlimbs were sectioned into three regions (Fig 1). Section “A” was made by cutting a cross-section on both sides of the ink, and placing the distal side down in a cassette. Section “B” was made by cutting 2 mm proximal to the stifle (knee) and placing the distal side down in the cassette. Section “C” was made by cutting 2 mm proximal to the hock (tarsal joint) and placing the cranial side down in the cassette (Fig 1). Then the individual sections were rinsed in their cassettes for 30 minutes. Samples were transferred to 70% ethanol, paraffin processed on an 8-hour schedule, and then paraffin embedded to maintain the same orientation obtained at grossing. Five paraffin sections were made at each of the 3 regions, separated by 100 μm. The first section was stained for hematoxylin and eosin (H&E) and the second for Masson’s trichrome. Images were captured from whole-slide images acquired with the Aperio ScanScope AT (Aperio, Carlsbad, CA, USA) using the 20x objective.

### Immunofluorescence

Immunofluorescence (IF) was conducted in *mdx* and control mice. The following muscles of the right hindlimb were harvested at the end of the study: tibialis anterior (TA); extensor digitorum longus (EDL); soleus (SOL); gastrocnemius (GA) (both the medial and lateral GA combined); and quadriceps (QUAD). Dissected muscles were embedded in Tissue-Tek OCT medium (Sakura Finetek USA, Torrance, CA) and frozen in liquid-nitrogen-cooled isopentane. Sections were cut transversely at the midbelly region in a cryostat at 10 μm thickness. Cryosections were blocked in KPBS (20 mM potassium phosphate pH 7.4, 150 mM sodium chloride) containing 0.3 mg/ml bovine serum albumin (BSA) and 1% Tween-20. The blocked sections were incubated for 2 hours with primary antibodies against the N-terminal region of dystrophin (raised in rabbit, used at 1:800) and the α2-chain of laminin (raised in rat; Sigma, St. Louis, MO, used at 1:800) in KPBS-G containing 2% normal goat serum. After several washes with KPBS-G, the sections were incubated for 1 hour with Alexa Fluor 488 goat anti-rabbit IgG and Alexa Fluor 594 goat anti-rat IgG (Molecular Probes, Eugene, OR, used at 1:1200). Immuno-stained slides were washed repeatedly with KBPS-G and mounted with ProLong Gold antifade reagent with DAPI (Molecular Probes, Eugene, OR). Images of the entire muscle cross-section were captured using a Nikon Eclipse E1000 fluorescent microscope (Nikon, New York, NY) attached to a digital camera with slightly overlapping fields to obtain images from the entire muscle. The cross-section of the muscle was reconstructed in Adobe Photoshop from the digital images. Quantitative analysis was performed by counting fibers in a minimum of four random fields (800 fibers per muscle cross-section). These fields were used to measure both myofiber areas and the percentage of myofibers with centrally located nuclei. The total area occupied by μDys-positive fibers was calculated as a percentage of the total muscle area. For bright-field microscopy, cryosections were briefly fixed in methanol and stained with Gill’s hematoxylin and eosin-phyloxine. The sections were washed, dehydrated and cleared in xylene before mounting with Permount (Fisher Scientific, Fairlong, NJ).

## Results

To provide verification that the treatment protocol resulted in improvements to myofiber morphology and muscle physiology we examined tissue sections of treated and control animals and performed *ex vivo* EDL muscle force measurements. Furthermore, micro-dystrophin (μDys) expression and serum levels for creatine kinase (CK) were used to assess efficacy of transgene delivery.

**Fig 2:**
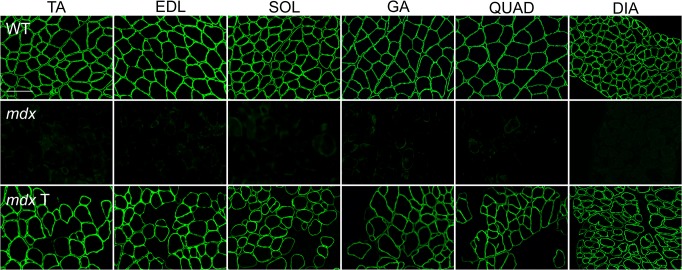
Whole-body micro-dystrophin gene transfer to the musculature using rAAV6. AAV6.CK8.H3μDys was administered by retro-orbital injection of 3-month-old *mdx* mice at a dose of 10^13^ vg, and tissues were analyzed 6 months later. All striated muscles were found to express widespread levels of the human micro-dystrophin. Shown are representative cross-sections of the TA, EDL, soleus, gastrocnemius, quadriceps, and diaphragm muscles immunostained with a rabbit polyclonal antibody against the N-terminal domain of dystrophin. Top row, muscles from wild-type mice; middle row, muscles from *mdx* mice; bottom row, muscles from *mdx* mice injected with rAAV6.CK8.H3μDys. Scale bar: 100 μm.

**Fig 3:**
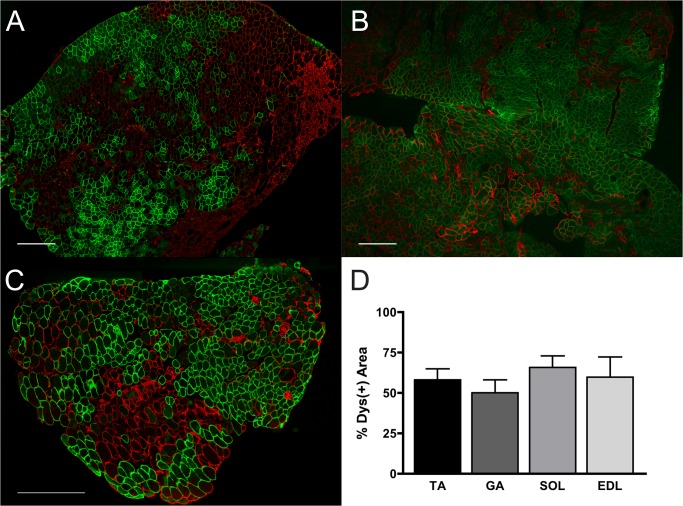
Anti-dystrophin staining for TA, GA and SOL muscles. Anti-dystrophin sAnti-dystrophin staining (green) shows clusters of transduced myofibers in representative sections of (A) TA, (B) GA, and (C) SOL muscles. Negative fibers (red) revealed morphological features of necrosis. (D) Between 50 to 65% of the total cross-sectional myofiber area of the selected muscles stained positive for dystrophin (*n* = 4). Scale bar: 500 μm.

We observed widespread expression of the transgene in the limb and diaphragm muscles of 9-month-old *mdx* mice following treatment with 10^13^ vg of rAAV6.CK8.H3μDys by retro-orbital (RO) injection at 3 months of age (Fig 2). Although dystrophin expression was observed in all muscles examined, positive fibers were distributed in clusters covering approximately 50–65% of the total cross-sectional area of individual muscles (Fig 3). A modest reduction in serum CK levels from 12,000 to 8,000 U/L was also observed.

**Fig 4:**
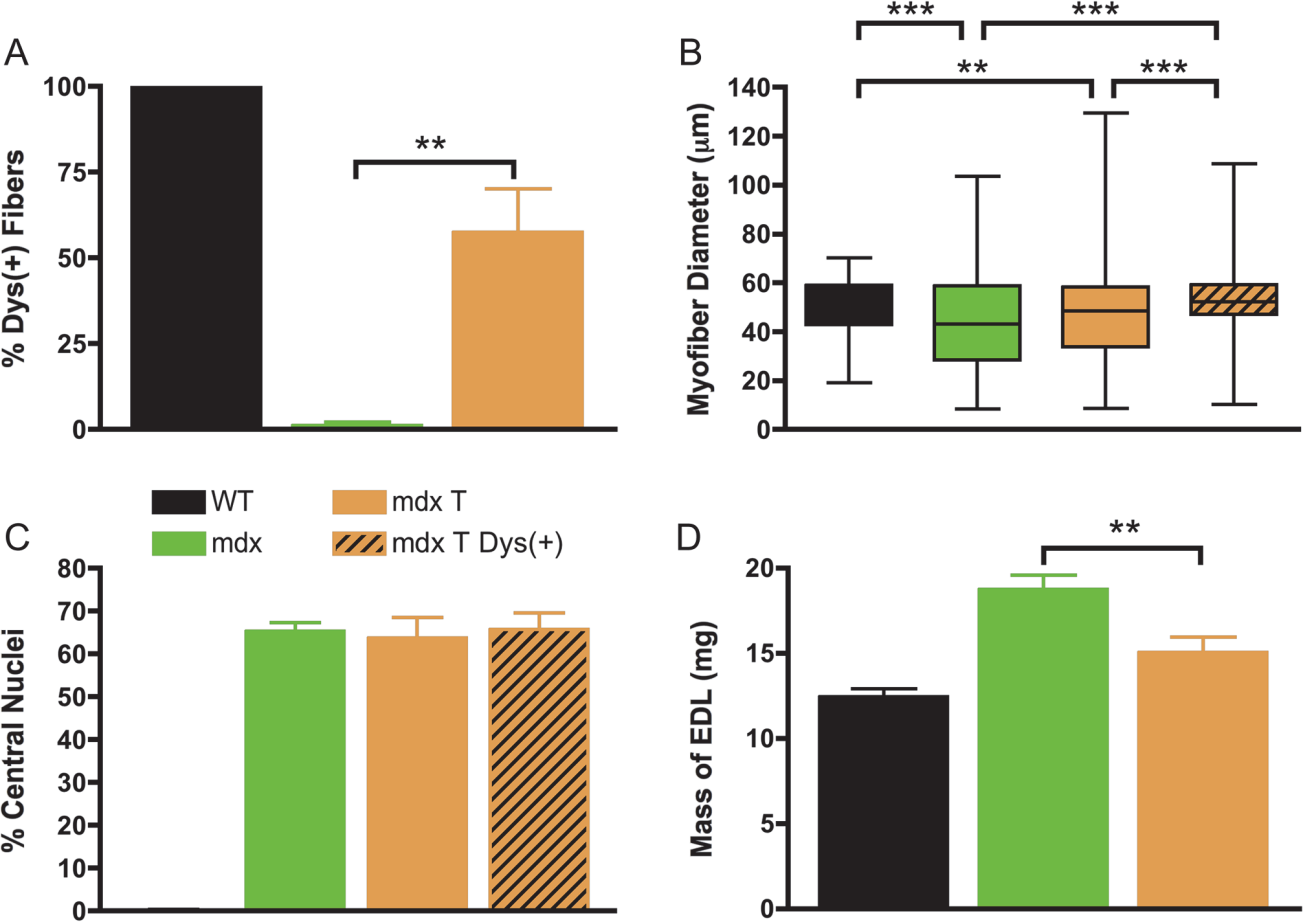
Systemic administration of rAAV6-μDys enhances the structural properties of EDL muscles in dystrophic mice. (A) Treatment restored expression of dystrophin in the majority of EDL myofibers of treated mice, in contrast with no expression in the muscles of untreated mice and complete expression in the muscles of wild-type mice. (B) Dystrophin-positive myofibers of treated EDL muscles (mdx T Dys(+)) were larger in diameter (median myofiber diameter; wild-type, 54 μm; dystrophic, 43 μm; treated dystrophin-positive fibers, 52 μm). Shown is the mean and distribution (25th and 75th percentile are represented by the box, and the whiskers represent the farthest diameter of muscle fibers). (C) There was no change in central nucleation compared with the myofibers in the muscles of untreated mice. (D) EDL muscle mass was restored to wild-type values in treated dystrophic mice. *n* = 3 for WT; *n* = 3 for *mdx*; and *n* = 4 for *mdx* T. Error bars represent SEM. *P < 0.05, **P < 0.01, and ***P < 0.001.

**Fig 5:**
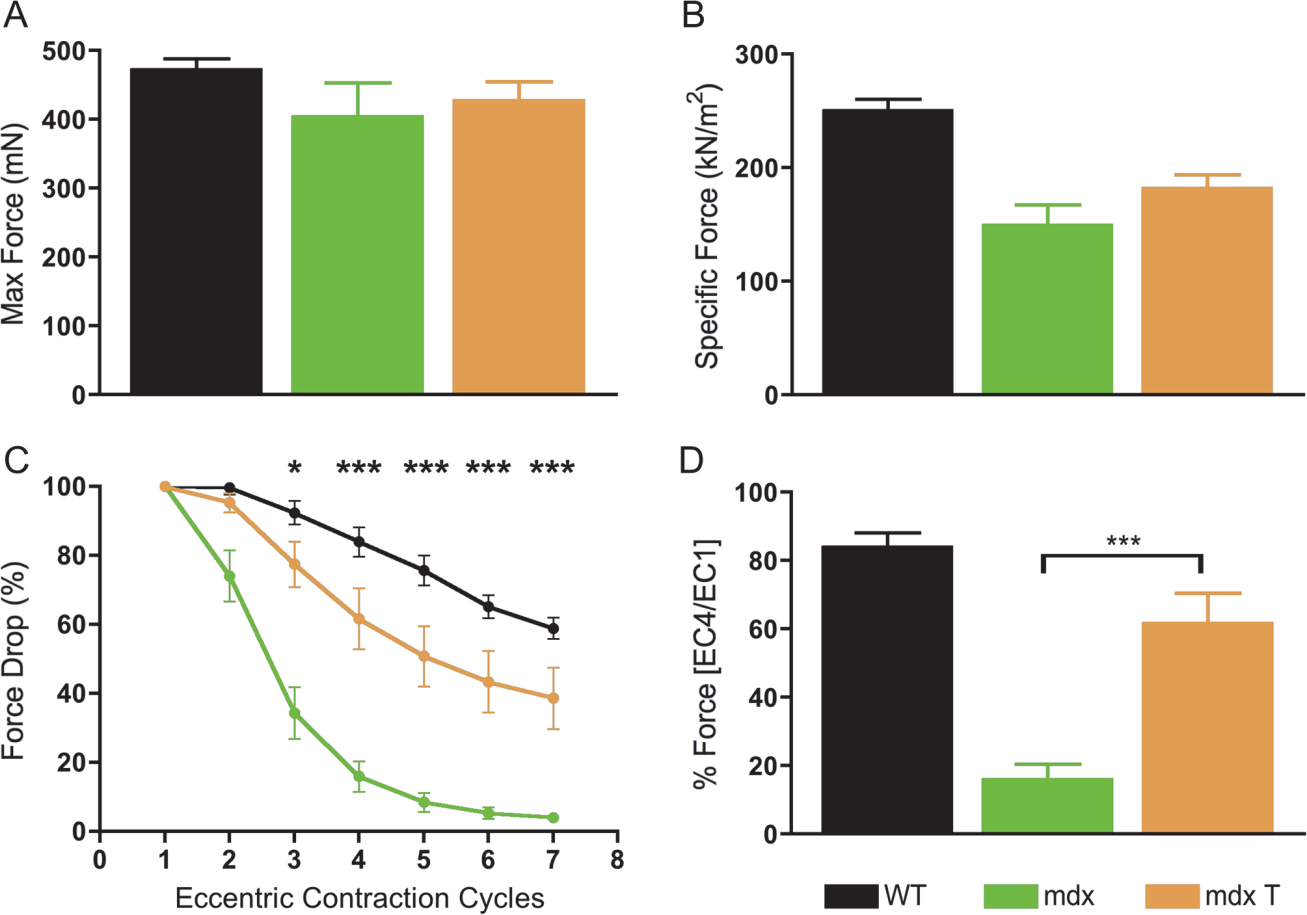
Systemic administration of rAAV6-μDys improves some functional properties of EDL muscles in dystrophic mice. (A) The maximum peak isometric force-producing capacity of EDL muscles was unchanged among wild-type, untreated, and treated mice. (B) Treatment did not improve the peak isometric-force-producing capacity of EDL muscles as normalized for cross-sectional area (wild-type, 250 ± 10; *mdx*, 149 ± 18; treated *mdx*, 182 ± 12 kN/m^2^). (C) The muscles of treated mice show improved resistance to contraction-induced injury when subjected to consecutive eccentric contractions of 20% beyond optimal muscle length. Bars represent the mean ± SEM percentage of the initial optimal muscle contraction. Treated muscles were significantly (*P < 0.05; ***P < 0.001) protected from contraction-induced injury when compared to *mdx* mice. (D) A comparison of the fourth versus the first contraction demonstrates response to injury following treatment was significantly improved over untreated *mdx*. *n* = 7 for WT; *n* = 8 for *mdx*; and *n* = 4 for *mdx* T. Error bars represent SEM.

**Fig 6:**
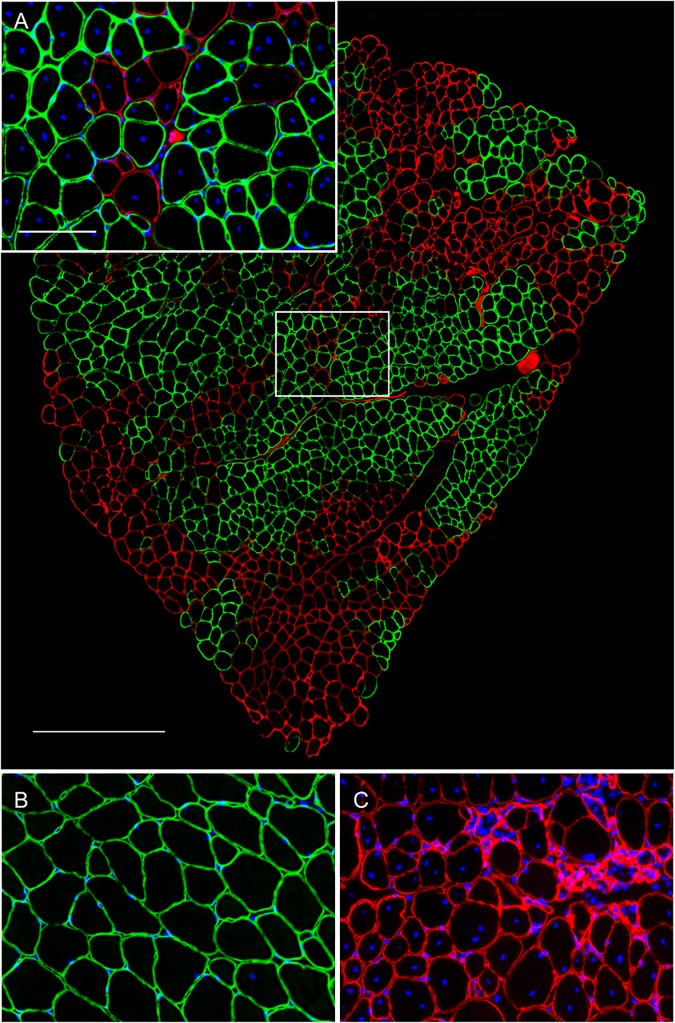
Immunofluorescent staining of 9 month EDL muscle for laminin (red), dystrophin (green), and nuclei (blue). (A) A composite cross-sectional image of an EDL muscle from a treated *mdx* mouse and a detail of the boxed area. (B) Control C57BL/6 EDL muscle sections demonstrate normal sarcolemmal localization of dystrophin and normal morphology. (C) Staining of EDL sections from *mdx* mice reveals the absence of dystrophin and demonstrates morphological characteristics of dystrophy, including variation in fiber size and abundant centrally located myonuclei. Scale bars: 100 μm (A-C); 1 mm (composite image).

The transduction and muscle contractile profiles in the EDL muscles were evaluated in-depth (Figs 4 and 5). We found extensive μDys expression (57 ± 12%) in the EDL muscle of treated *mdx* mice (Figs 6A and 4A) six months after treatment. Fiber size variation and centrally nucleated fibers in *mdx* mice are consistent with ongoing muscle damage and regeneration. Histological evaluation of the treated group revealed a significant increase in fiber size to that of normal mice, and a decrease in size variability in the dystrophin positive myofibers. Comparing *mdx* controls with treated *mdx*, the average myofiber diameter increased from 44.1 to 52.5 μm (Fig 4B, *P* < 0.001). At ~9 months of age, 65 ± 3.5% of the untreated *mdx* EDL muscle myofibers contained centrally located nuclei. No reduction in the percentage of central nucleation in myofibers of treated *mdx* mice was observed, possibly due to repeated cycles of regeneration prior to treatment at 3 months of age (Fig 4C). Although myofiber transduction was significant, efficiency may have been reduced in 9-month-old *mdx* mice due to the increased fibrosis and muscle fiber damage that would have been present in 3-month old *mdx* muscle at the time of treatment.

Mice lacking dystrophin are more susceptible to contraction-induced injury. Upon termination of the study, we isolated the right EDL from each mouse and performed *ex vivo* force measurements to test how expression affected muscle contraction. Comparing absolute isometric tetanic force between *mdx* mice, we found treatment with rAAV6.CK8.H3μDys resulted in a small improvement compared with untreated *mdx* mice, however differences did not reach statistical significance, and they were not significantly different than wild-type values (Fig 5A). To assess the specific force, we normalized per cross-sectional area (CSA) and found AAV6-mediated H3μDys therapy improved function compared with untreated *mdx*, but did not reach statistical significance (*P* > 0.05) (Fig 5B) and both were below wild-type animals. We next subjected each EDL to a series of repeated eccentric contractions. Comparing decay curves, AAV-mediated H3μDys expression provided protection against eccentric contraction-induced injury (Fig 5C). We saw statistically significant improvements following the second eccentric contraction (*P* < 0.001). A comparison of the fourth versus the first contraction demonstrates that response to injury following treatment was increased over untreated *mdx* (Fig 5D). Thus, treatment with rAAV6.CK8.H3μDys provides an overall improvement in resistance to eccentric contraction-induced injury, although it does not restore specific force generation to wild-type levels.

Having established a comprehensive profile of the histological features and functional changes of *mdx* skeletal muscle treated with rAAV6.CK8.H3μDys, our focus will be on the applicability of MRI as a tool to monitor disease progression and efficacy of treatment, starting with an interpretation of T2 values and how they compare to muscle histology.

**Fig 7:**
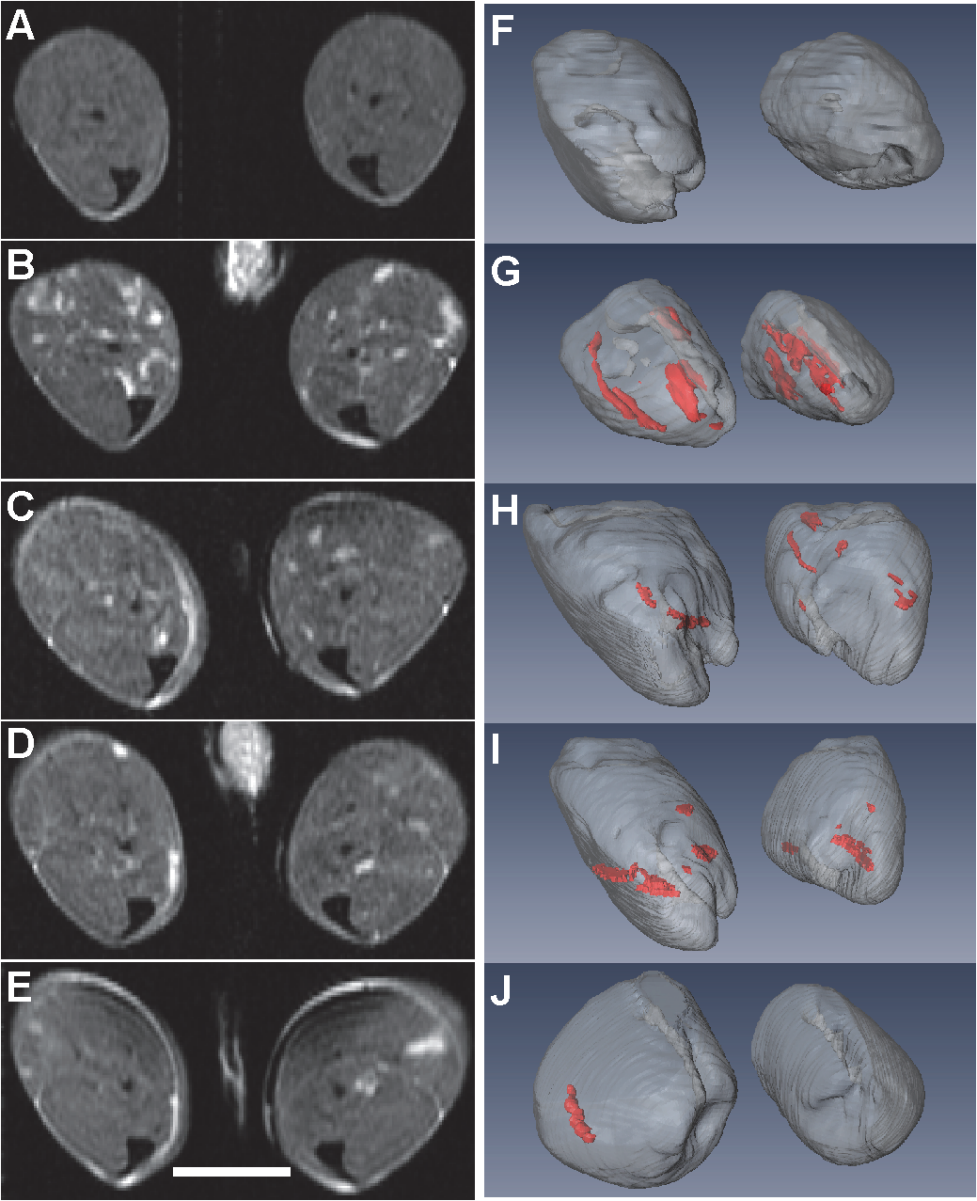
3D visualization of longitudinal T2w imaging. T2w images for a normal mouse (A) and an *mdx4cv* mouse before (B) and 8 weeks (C), 16 weeks (D) and 24 weeks (E) after AAV vector mediated gene therapy. 3D muscle volume with necrotic regions segmented with high resolution in red. 3D T2w image acquired for the same normal mouse (F) and the four time points (G-J) for the *mdx4cv* mouse. Scale bar = 5 mm.

Fig 7 displays T2w images alongside 3-D rendered muscle volumes with necrotic regions highlighted in red. These regions appear as hyper-intense on the T2w images. T2w images for a normal mouse (A) and an *mdx* ^*4cv*^ mouse before (B) and 8 weeks (C), 16 weeks (D) and 24 weeks (E) after AAV vector-mediated gene therapy highlight the changes in the musculature—T2 values were measured on unaffected muscles for TA (K), GA (L) and SOL (M) (hyperintense areas avoided). To visualize the effects of the therapy or natural necrosis/regeneration, we created 3D muscle volume renders with necrotic regions segmented in red using high-resolution 3D T2w images (the comparison being between a normal mouse (F) and the four time points (G-J) for an *mdx* ^*4cv*^ mouse). With the application of the gene therapy, we would expect the treated mice to display less necrosis or other pathological regions in the tissue. The visual results indicate an overall decrease in the affected regions post-treatment for the treated mice.

Table 2 displays the measurements for each group, separated by muscle type and time point. All values had % change values calculated by taking the difference of the two data points and dividing by the initial value. Once this was calculated for each individual mouse, the values for each group were averaged to produce group values (treated and untreated). The T2 values showed an overall larger percentage decrease: -11.7% versus (vs) -4.7%, -8.2% vs -6.2%, and -7.1% -7.0% for the TA, GA, and SOL muscles, treated vs untreated, respectively in the values measured when comparing the pre-treatment values to Post-Rx 24 week values. The ADC values measured showed a higher range of variability, with the TA muscle measurements: -5.3% vs -1.9% while the GA and SOL muscles: 3.3% (an increase in value) vs -0.4% and -1.7% vs 0.2%. The MT Ratio values also showed percentage changes between the two groups of animals (-8.9 vs -4.0, -11.8 vs 0.14, and -6.8 vs -3.9 for treated vs untreated TA, GA, and SOL muscles, respectively).

**Table 2:** Values measured of MR parameters for each group, separated by muscle type and imaging time point.

MR methods	Muscle	Treatment	Pre-Rx	Post-Rx 8wk	Post-Rx 16wk	Post-Rx 24wk
**T2 (ms)**	TA	No	20.4 ± 1.1	19.4 ± 0.7	19.4 ± 0.5	19.0 ± 0.1
		Yes	20.7 ± 1.3	18.8 ± 0.4	18.9 ± 0.3	18.8 ± 0.4
	GA	No	21.3 ± 1.4	20.0 ± 0.1	19.6 ± 0.1	20.3 ± 0.2
		Yes	21.0 ± 1.1	19.5 ± 0.7	19.3 ± 0.3	19.9 ± 0.3
	SOL	No	20.8 ± 0.4	19.7 ± 0.3	19.8 ± 0.4	19.6 ± 0.3
		Yes	20.5 ± 0.4	18.9 ± 0.4	18.9 ± 0.2	19.2 ± 0.1
**ADC (x 10^–5^ mm^2^/s)**	TA	No	135 ± 5	132 ± 3	143 ± 11	129 ± 4
		Yes	137 ± 5	139 ± 9	139 ± 5	130 ± 6
	GA	No	140 ± 7	132 ± 3	132 ± 11	140 ± 4
		Yes	139 ± 5	140 ± 9	146 ± 5	143 ± 4
	SOL	No	133 ± 4	130 ± 5	132 ± 7	133 ± 5
		Yes	134 ± 4	135 ± 6	134 ± 4	133 ± 9
**MTR (%)**	TA	No	64.8 ± 4.1	62.8 ± 6.7	63.7 ± 5.0	62.4 ± 8.8
		Yes	64.7 ± 3.8	62.2 ± 1.9	58.9 ± 3.3	57.9 ± 4.9
	GA	No	63.1 ± 3.1	60.2 ± 9.5	64.5 ± 6.2	63.3 ± 3.8
		Yes	62.9 ± 4.0	58.4 ± 6.3	58.2 ± 3.5	54.8 ± 7.4
	SOL	No	63.6 ± 3.0	62.8 ± 7.2	62.7 ± 6.7	61.7 ± 8.7
		Yes	63.3 ± 3.0	62.3 ± 1.4	59.3 ± 3.0	57.8 ± 4.6

Values for each muscle type were averaged within the respective treatment and non-treatment groups and then compared at each time point.

**Fig 8:**
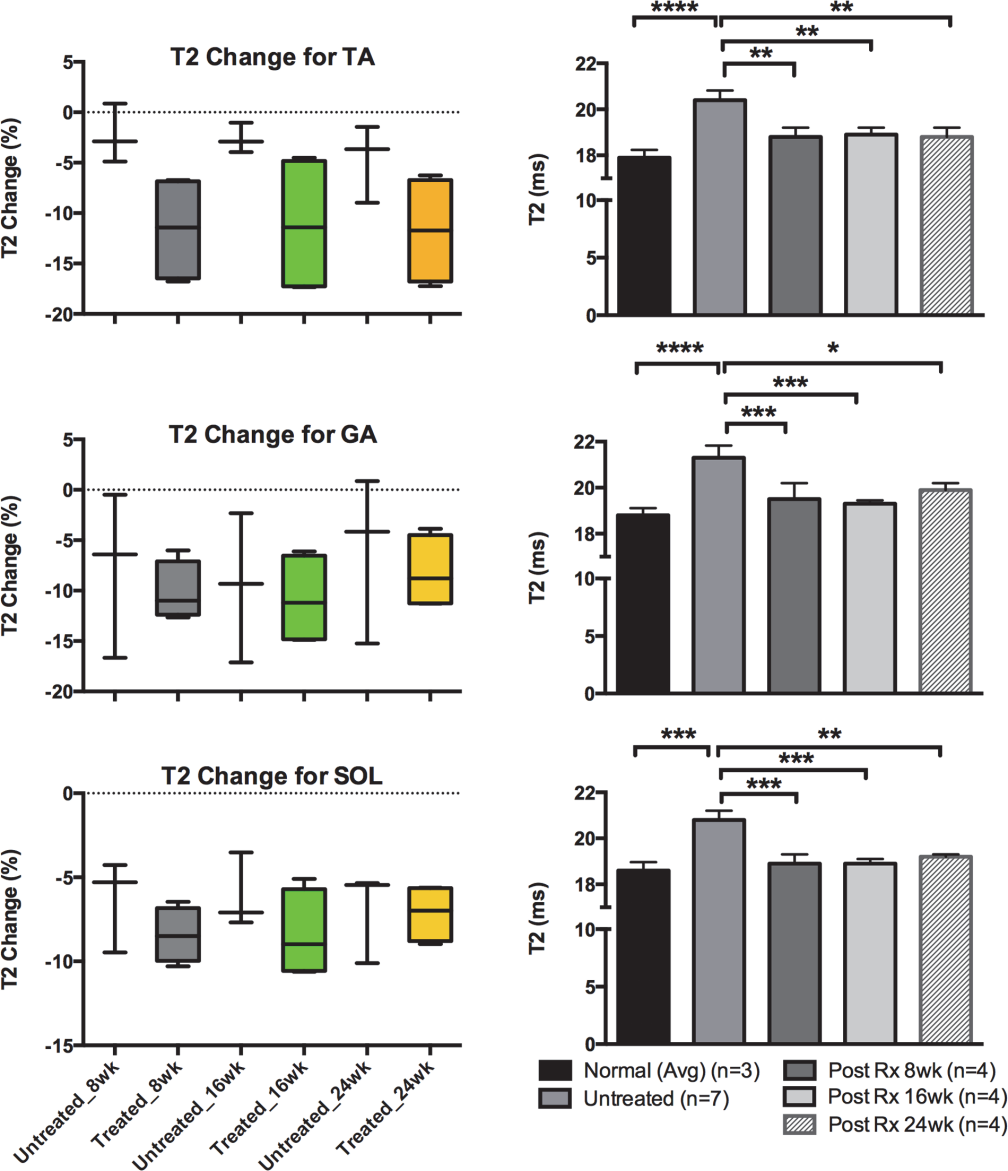
T2 values analyzed for TA, GA and SOL muscles. Graphs for the quantified image values taken from the T2 modality for different muscle types of TA, GA and SOL muscles—all images were reviewed and measured. The graphs on the left column depict average T2% change values for both treated and untreated *mdx* mice at each time point for each muscle type while the graphs on the right display averaged T2 values for all mice including the normal (the normal mice had both pre and post time point data values averaged). *P < 0.05, **P < 0.01, ***P < 0.001 and ****P < 0.0001.

**Fig 9:**
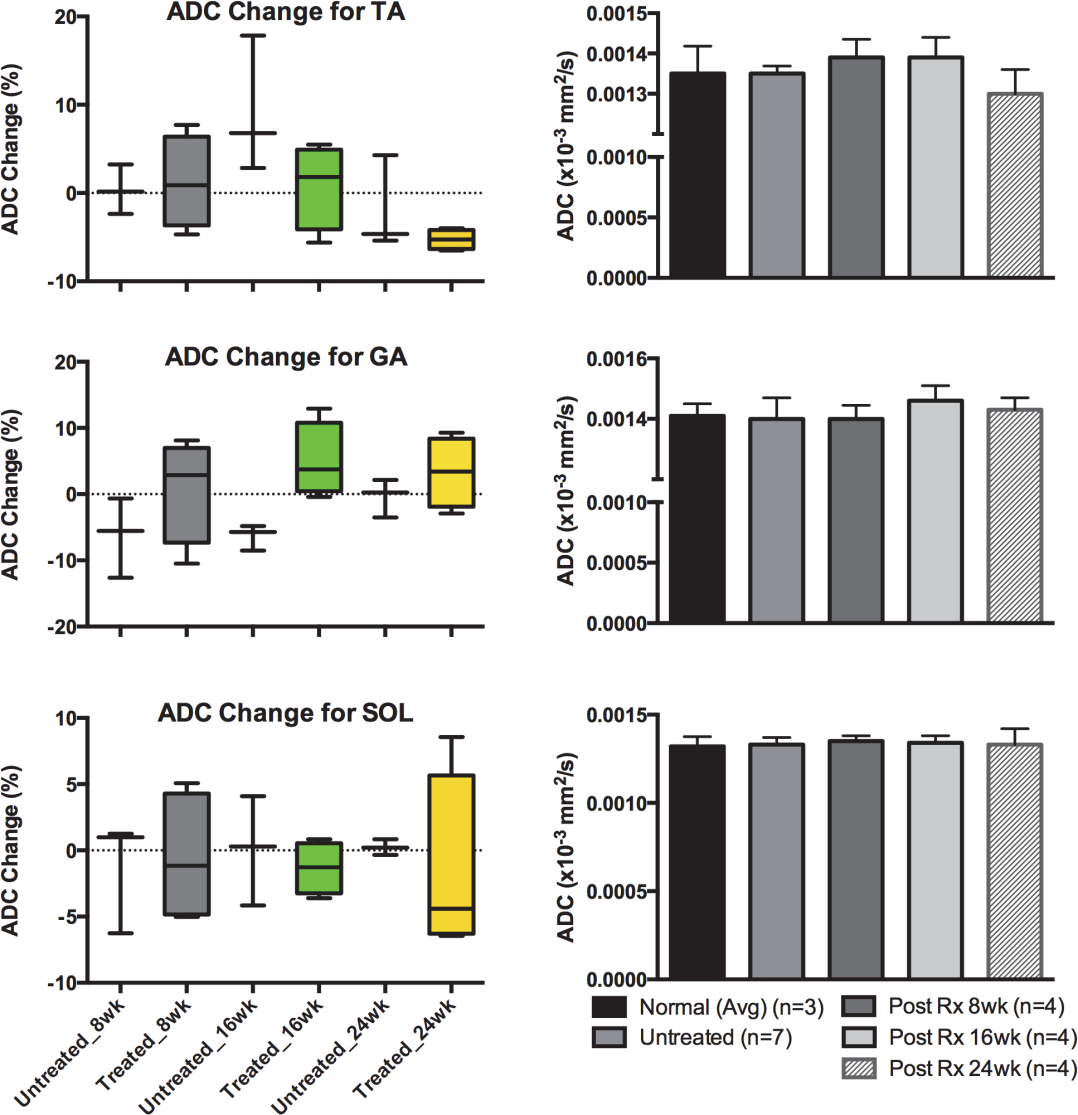
ADC values analyzed for TA, GA and SOL muscles. Graphs for the quantified image values taken from the ADC modality for different muscle types of TA, GA and SOL muscles—all images were reviewed and measured. The graphs on the left column depict average ADC % change values for both treated and untreated *mdx* mice at each time point for each muscle type while the graphs on the right display averaged ADC values for all mice including the normal (the normal mice had both pre and post time point data values averaged).

**Fig 10:**
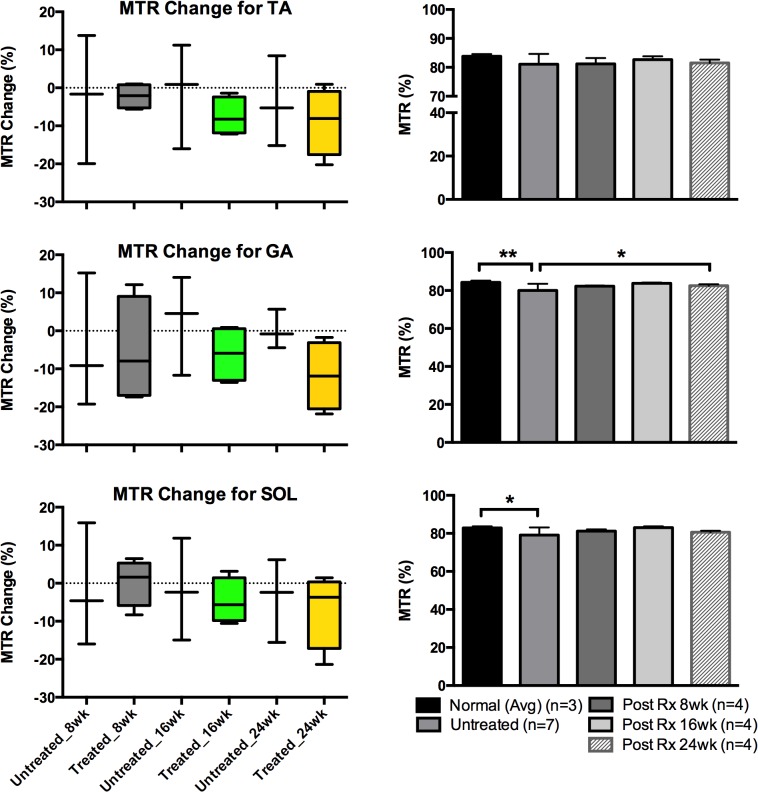
MTR values analyzed for TA, GA and SOL muscles. Graphs for the quantified image values taken from the MTR modality for different muscle types of TA, GA and SOL muscles—all images were reviewed and measured. The graphs on the left column depict average MTR % change values for both treated and untreated *mdx* mice at each time point for each muscle type while the graphs on the right display averaged MTR values for all mice including the normal (the normal mice had both pre and post time point data values averaged). *P < 0.05.

Figs 8–10 display the measurements of comparing untreated vs treated groups in terms of % changes for each imaging modality on the left based off the data from Table 2, while the right displays the progression of average values measured for the treated mice (n = 4) against the initial average of both the normal (n = 3) and all *mdx* ^*4cv*^ mice (n = 7). Note the levels of significance for the T2 are more prominent in all 3-muscle groups of TA and GA and SOL muscles. These measurements were to investigate the differences between each group at any given time point.

The statistical analysis was carried out via two-way ANOVA that compared columns within rows and accounted for simple effects within rows: the columns were time points (Pre-Rx and 8, 16, 24 weeks Post-Rx) for the various groups while the rows were the 3 different muscle groups (TA, GA, and SOL). The analysis showed similar trends: the T2 measurements were significant for all three muscle types in regard to Pre-Rx (treatment) measurements vs Post-Rx measurements, along with the GA and SOL displaying significance between the untreated and treated groups. The *P*-value for the comparison of the normal and all *mdx* mice Pre-Rx was < 0.0001, for the TA and GA muscles, while the SOL had a *P*-value of 0.0001. The TA, GA, and SOL muscle groups continued to show significance between the untreated and treated groups for all subsequent time points in the T2 modality: 0.0018, 0.0043, and 0.0018 for the 8, 16, and 24 weeks Post-Rx, respectively (TA), 0.0003, 0.00001, and 0.0097 (GA), and 0.0001, 0.0001, 0.0018 (SOL). The MTR measurements for the same muscle groups also showed significance in several muscle groups: 0.026 (SOL in normal vs untreated mdx), 0.024 (GA in normal 24 week Post-Rx vs untreated), and 0.0062 (GA in normal mice averaged pre and post Rx vs untreated).

**Fig 11:**
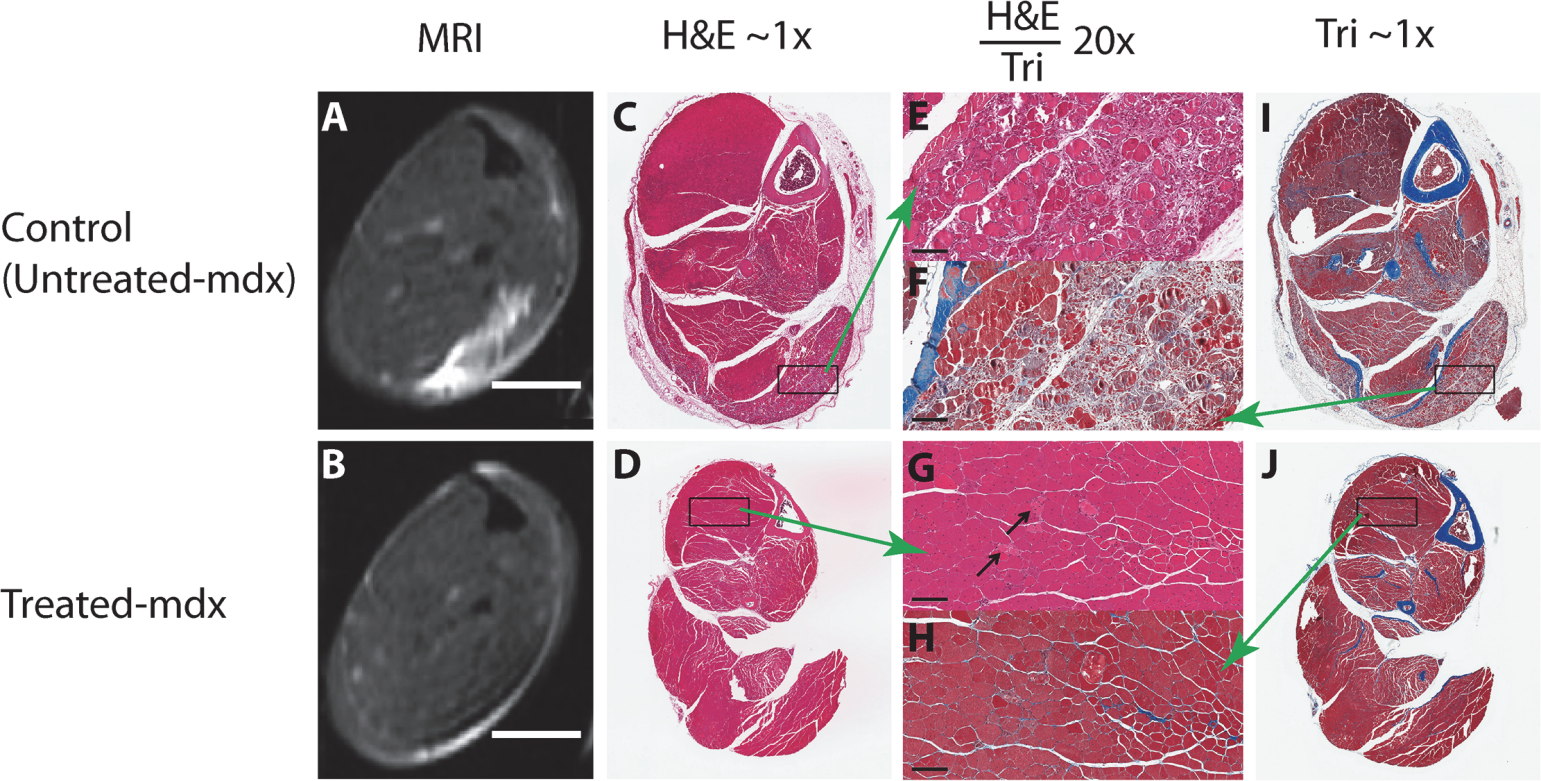
Tissue Sample Analysis. Histology was done on the left hindlimbs of all mice involved in the study. Representative T2 images are present (A) and (B). (C) Representative H&E images of an untreated *mdx* mouse at 1.6x and (E) is a 20x magnification of the gastrocnemius (GA) muscle (boxed area in (C)). (I) Representative Masson trichrome staining from an untreated *mdx* mouse at 1.6x and (F) is a 20x magnification of the GA muscle (boxed area in (C)). (D) Representative H&E images of a treated *mdx* mouse at 1.6x magnification and (G) is a 20x magnification of the tibialis anterior (TA) muscle (boxed area in (D)). (J) Representative Masson trichrome staining from a treated *mdx* mouse at 1.6x magnification and (H) is a 20x magnification of the TA muscle (boxed area in (J)).

The MRI measurements taken were correlated with histopathology (utilizing H&E and Masson’s trichrome staining) to visually represent the cellular processes of muscle degeneration and regeneration (Fig 11). We attempted to utilize histology as a means of corroborating what was seen in various MR techniques, such as muscle necrosis and fibrosis. The hyperintense regions visible in the T2 images, for example, can be seen as necrotic tissue in the H&E stained histology slides. Fig 11 displays tissue sample analysis: histology was done on the left hindlimbs of all mice involved in the study. Representative T2 images are present (Fig 11A) and (Fig 11B) for untreated and treated *mdx* mice, respectively. The untreated *mdx* mouse in Fig 11A shows a large hyper-intense region situated near the gastrocnemius (GA) muscle. No such region is visible in the treated *mdx* mouse as displayed in Fig 11B. Fig 11C and 11E show representative H&E images of an untreated *mdx* mouse at 1.6x and 20x magnification of the GA muscle, respectively. The untreated *mdx* mouse showed variability in fiber size with multifocal myofiber necrosis, mononuclear cellular infiltrate, and increased connective tissue (fibrosis). Multifocal small fibers with centralized nuclei (regenerative) are seen in the periphery. Fig 11I and 11F display representative Masson trichrome staining from an untreated *mdx* mouse at 1.6x and 20x magnification of the GA muscle, respectively. Collagen stains blue with trichrome stain in the Masson trichrome stained images. There is increased fibrous connective tissue noted in the untreated *mdx* mouse compared to the treated *mdx* mouse. Fig 11D and 11G depict representative H&E images of a treated *mdx* mouse at 1.6x and 20x magnification of the tibialis anterior (TA) muscle, respectively. There are multifocal necrotic muscle fibers (arrows) surrounded by smaller fibers with centralized nuclei (regeneration). There is minimal mononuclear infiltrate. Fig 11J and 11H show representative Masson trichrome staining from a treated *mdx* mouse at 1.6x and 20x magnification of the TA muscle, respectively. There is a reduction in fibrous connective tissue in the treated *mdx* mouse compared to the untreated *mdx* mouse.

## Discussion

The success of gene therapy via RO injection of a codon-optimized micro-dystrophin gene was evaluated through a histopathological examination of skeletal muscle fibers in conjunction with MRI to monitor treatment, as well as a physiological assay to assess muscle function. We first examined central nucleation in transduced myofibers and found that in the older *mdx* myofibers the number of centrally-located nuclei remained unchanged, in both dystrophin negative and positive fibers. However, anti-dystrophin staining of the TA, GA, and SOL muscles in *mdx* mice revealed clusters of transduced myofibers that were of more normal uniform structure, whereas the dystrophin negative regions revealed features of necrosis consistent with muscle damage and regeneration. Furthermore, we found that micro-dystrophin expression in approximately half of the myofibers was sufficient to protect EDL muscle from contraction-induced injury. Only a marginal improvement in specific force production was attained in the treated *mdx* EDL muscle. This may have been due to suboptimal transduction efficiency at the time of treatment in the 3-month *mdx* mice. Data derived from MRI measurements correspond to histological indices indicative of muscle damage, and can be used to monitor the success of a therapeutic treatment and course of disease.

MRI has been shown to be a useful tool for the investigation of skeletal muscular damage, functionality, and regeneration. In particular, T2 measurements have been used in a variety of muscle-tissue based investigations—measurements based on the T2 weighted (T2w) modality seem to be both sensitive and consistent. Our work looked to expand the non-invasive MR toolset available in the assessment and treatment of muscular dystrophy. Because the different modalities are sensitive to distinct properties of the muscle tissue, it is a practical and necessary step to investigate the refinement of possible MR tools for the assessment of muscular dystrophy.

In general, the T2 relaxation of skeletal muscle is composed of at least three components with values < 5, 25–45 and > 100 ms, where the intermediate value range constitutes most of the signal [40–43]. These T2 values have been attributed to the hydration of macromolecules, intracellular and extracellular water, respectively. In this study, we used echo times ranging from 6 to 100 ms that can mostly identify responses for both the intracellular and extracellular water. In addition, our T2 quantification used the fat suppression method to minimize T2 contribution from fat to muscle T2. Therefore, T2 increase in this study may reflect an increase of extracellular compartment and/or an increase of necrosis or an increase of inflammation/edema in skeletal muscle. Diffusion weighted MRI in this study was used to determine the self diffusion of water in tissue, which is affected by the presence and orientation of physical barriers. Cellular damage alters the structural organization resulting in changed diffusion [44]. ADC values determined by diffusion weighted MRI did not show significant changes among normal, treated and untreated muscles. This is probably due to the fact that myocellular changes may be small in the *mdx* ^*4cv*^ mouse model at the ages for our study. MT measurements showed some statistical significance between the normal and untreated *mdx* ^*4cv*^ mice in their GA and SOL muscles. Magnetization transfer ratio values are sensitive to macromolecular changes in muscle such as formation of fibrosis. We do not expect a large formation of fibrosis in the adult *mdx* ^*4cv*^ mice used in this study. However, our MT results show the potential formation of fibrosis in GA (mixed twitch) and SOL (slow twitch) muscles, demonstrating the MTR method would be sensitive to macromolecular changes in skeletal muscle. TA muscle, comprising fast twitch fibers, in the *mdx* ^*4cv*^ mouse model did not show a sign of macromolecular changes.

According to this analysis, the T2 results show the most robust response between both treatment and time point. The two-way ANOVA looking into the treatment groups show strong significance in the same pattern: the T2 *P*-values show high significance between the pre and post-treatment values in all muscle types (as seen in bar graphs of Figs 8–10). Post-treatment values in the T2 modality were comparable to the values measured in the normal mice. This is also supported by the % change values (box graphs of Figs 8–10) for each timing point. These values indicate the initial differences between values for the untreated and treated animals and the trends that emerged as the data was collected. In conjunction with the histopathology and imaging comparisons, the results consistently indicate the continuing usefulness of T2 in longitudinal treatment/pathology tracking. While the ADC and MTR measurements may not have shown as much statistical significance in the ANOVA, it is still worth noting the values of the data collected for all the imaging modalities show a decreasing trend in the values observed with only one exception. Also, it is noted that T2 values were decreased after 10 weeks of age regardless of treatment. This finding is similar to what Pratt *et al*. found in their longitudinal T2 measurements conducted with one *mdx* mouse from 5 weeks of age to 80 weeks of age [45].

The decrease in T2 values measured for post-treatment, alongside the significance in statistical measurements, indicates that T2 quantification can act as a good MR marker in non-invasive assessment and evaluation of therapeutic treatment. Although T2 values changed in response to treatment and correlated with histopathology, a more precise correlation would require refined MRI protocols and identification of specific cells types. Furthermore, an exact spatial correspondence between histology and MRI slices would be necessary to verify MRI findings. There is also an indication that magnetization transfer, and diffusion to an extent, can also be a useful MR modality with some further refinement in the sequence setup and protocol. Most notably, an investigation into a more refined diffusion measurement seems to be a good source of future potential. Further investigation is necessary to acquire images of higher quality (signal to noise ratio) and consistency with minimization of potential motion artifacts.

The present study itself was fairly narrow in its design and scope—there are several limitations. Because we are working with such small animals that must be anesthetized, there is a limited window of opportunity to acquire all the scans in the protocol before the mouse’s condition deteriorates. Thus, the MRI pulse sequences had to be modified in a way to yield the clearest images without being excessively long. This may have reduced the quality of some of the MRI maps, notably the diffusion maps that had the longest sequence time. Second, the time scale for the study itself may not have been the most optimal to give us the most robust results possible in terms of identifiable bio-markers in post MR analysis and histology. Notably, the study design might have missed the window for reported onset of progressive muscle replacement by fibrous tissue in younger *mdx* mice (10–13 weeks) and the extensive fibrosis/calcification seen in much older *mdx* mice (16–20 months) [46, 47]. The study began with the mice at 3 months of age; it would have been interesting to track possible incursion of fibrosis through several pre-treatment scans to compare treatment vs non-treatment for both very young mice and very old mice. Nevertheless, 3-month old *mdx* muscle may better represent the severe dystrophic pathology in human muscular dystrophy patients who may be less responsive to gene therapy.

Another limitation would be the random nature of the naturally occurring muscle degeneration and necrosis. Without a set exercise or induced-injury protocol, the study itself was not designed to study all the conditions under which the gene therapy could be administered, and consequently, be observed and correlated using MRI. As a baseline, the results show promise for future investigation that can be done utilizing more sophisticated and controlled observation.

In regard to histopathology and IF, the decalcification of the excised legs is an important component of specimen preparation for paraffin sections. Over-decalcification removes valuable tissue information and limits the amount of information that can be utilized using these methods. Thus, a careful sample preparation as described in the Materials and Methods section should be conducted to prevent an over-decalcification. Furthermore, although morphology is much better retained in paraffin sections, not all antibodies will work with the harsh fixation involved in their processing. Therefore, frozen sections of harvested muscles from the contralateral leg were utilized for IF experiments to determine the extent of micro-dystrophin expression. In addition, time of the histology was an important factor pertaining to the *mdx* model itself—certain time points may be better for different muscle pathologies occurring. Because the histology was only taken for one time point post-treatment, the histology was somewhat limited. Future studies could look to expand this corroboration between histology and MR by taking more frequent histology samples at all stages of the study/treatment time points, and from both legs taking into account the asymmetric muscle pathology and force previously reported in *mdx* mice [48].

Our treatment protocol resulted in dystrophin expression that led to improvements of muscle function and morphology, as well as significant changes in MRI parameters, especially T2. Using MRI to examine T2 changes before and after treatment allows for repeated and longitudinal measurements to be made from the lower hindlimb muscles in a single animal. The ability to examine several muscles simultaneously is particularly useful, because not all muscles respond in the same way to treatment. Therefore, MRI may be used to complement histological and physiological studies by determining which muscles undergo the most damage and are more responsive to treatment.

In conclusion, longitudinal parametric MRI utilizing the T2 has feasibility in the identifying and monitoring the disease progression and treatment response in the *mdx* mouse model of muscular dystrophy. MT and diffusion effects will require further testing and refinement to fully assess their usefulness as MR techniques for muscular dystrophy disease and treatment tracking. While the T2 measures showed some statistical significance, the MT and diffusion effects did not show significant differences between treated and untreated muscles in the mdx mouse model. Consequently, MRI should prove useful in evaluating disease course and therapeutic efficacy in many muscular disorders such as muscular dystrophy.

## Supporting Information

S1 Dataset Animal information. (XLSX)
